# Quality control of the peer-review process by *Forensic Sciences Research*

**DOI:** 10.1080/20961790.2019.1700886

**Published:** 2019-12-29

**Authors:** Shiying Li

**Affiliations:** Editorial Office of Forensic Sciences Research, Key Laboratory of Forensic Medicine, Shanghai Forensic Service Platform, Academy of Forensic Science, Shanghai, China

With the rapid development of interdisciplinary studies in recent decades, science and technology has achieved both greater breadth and greater specialization, and the number of manuscripts published annually has increased year on year [1]. Studies that are established on the basis of integrity and credibility, and generate repeatable results that advance the development of a discipline can be considered the “best things”. As David Hume has put it, however, “The corruption of the best things gives rise to the worst” [2]. Some scientists may be motivated to pursue their own best interests in the short term, and an eagerness for rapid success and/or benefit in this context can evidently have negative effects. This situation is becoming serious. In 2015, a fake peer-review process was reported by Springer, and it caused 107 papers to be retracted by *Tumor Biology* [3]. Springer was not the first major publisher to discover widespread problems with the peer-review process that ultimately prompted mass retractions. BioMed Central retracted 43 papers in one fell swoop in November 2014, and in 2015 the Hindawi Publishing Corporation announced that three editors had “subverted” the entire editorial process involved in the publishing of 32 papers [4–8].

One reason for the aforementioned mass retractions was that a third-party agency authorized by the authors submitted the papers to journals for consideration for publication [8]. That third-party agency recommended reviewers to the journals’ editorial offices whose email addresses were fake and controlled by either the manuscripts’ authors, or the third-party agencies. In other words, these authors and/or agencies acted as both a player and the referee. The deception was facilitated when the journal editors chose the reviewers recommended by the third-party agency. Of course, email addresses located on the Internet by editors can also be fake. In general, the initial peer-review process includes three parts: preliminary reviewing by an editor, the sending out of reviewer invitations/requests (usually to two to three experts), then a decision being made by the journal’s editor-in-chief as to whether to accept or reject the manuscript for publication. Although some journals still invite authors to suggest peer reviewers for the manuscripts submitted to them, in recent years the potential perils of doing so have become more widely recognized by journals worldwide.

In view of the above-described potential pitfalls of the peer-review process in the era of digital academic communication, *Forensic Sciences Research* (*FSR*) has conducted a rigorous quality control assessment of the peer-review process it has utilised since the journal’s establishment approximately 3 years ago. The focus of the assessment was the potentially fake reviews/reviewers. During this assessment the *FSR* Editorial Office evaluated all peer reviewers of papers accepted and rejected from 12 December 2016 to 14 November 2019.

1. *FSR* received 352 submissions from the date of the journal’s establishment on 12 December 2016 to 14 November 2019, and to date final decisions (acceptance or rejection) have been made with regard to 291 of these manuscripts. For these 291 papers, 378 experts have completed 890 reviews and submitted their decisions, which include provisional acceptance pending revision, acceptance, or rejection. Of these experts, 39% were situated in Europe, 28% were situated in North America and the remainder were situated elsewhere (Figure 1).

**Figure 1.**
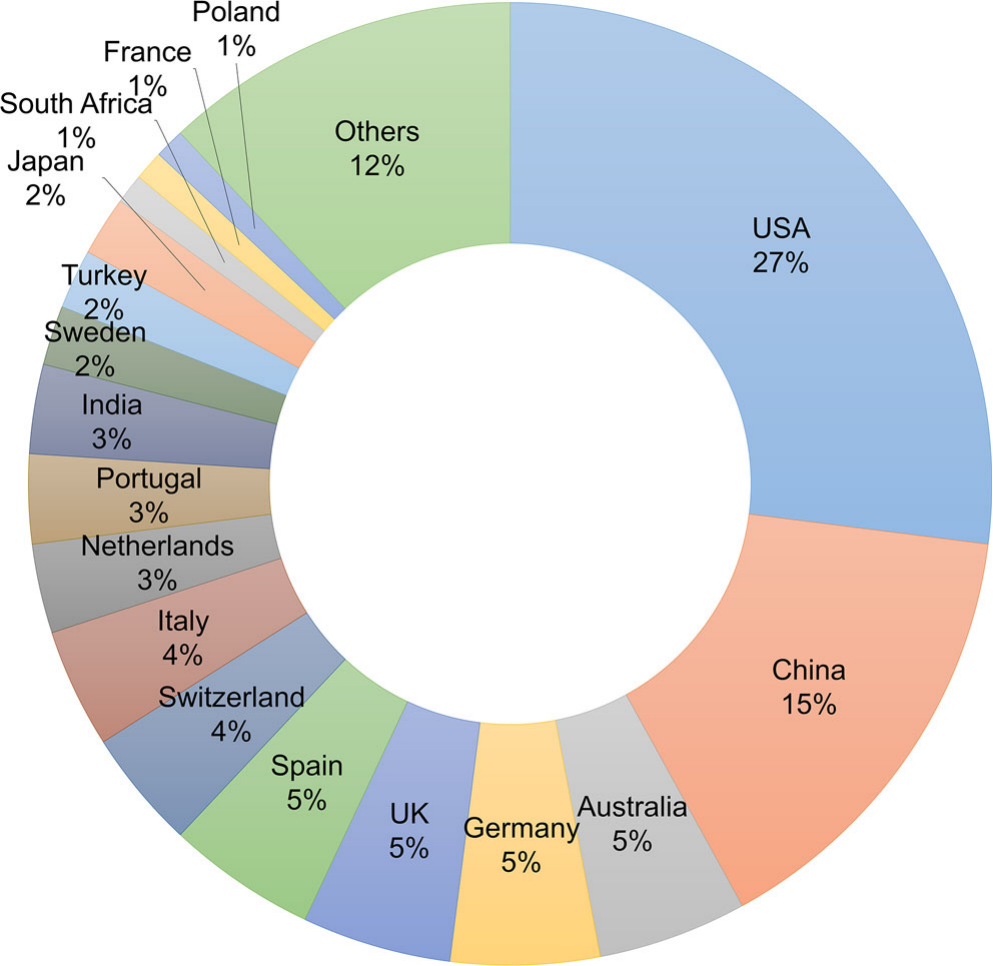
Geographic distribution of 378 experts invited to conduct peer reviews of manuscripts for Forensic Sciences Research from 12 December 2016 to 14 November 2019.
2. Of the 378 reviewers, 283 (75%) used institutional email addresses and the remaining 95 (25%) used non-institutional email addresses. The 95 non-institutional email addresses were all checked individually by using them to search for publications in which they were provided as corresponding author email addresses. At least one publication for each of these reviewers was located in this way, indicating that those email addresses were legitimately held by those reviewers.
3. The funding information included in all manuscripts published during the aforementioned ∼3-year period was also reviewed. Of 139 published manuscripts, 71 (51%) alluded to funding from the authors’ own affiliated institutions. To an extent, this result is indicative of the academic reliability of these papers.
4. In accordance with standard *FSR* processes, from 2016 to 2019 all peer reviewers were selected by *FSR* editors. Notably, for some invited papers in special issues some reviewers were recommended by guest editors because of their specific knowledge in a certain field. Nonetheless, all choices were based on the principle of double-blind peer review and were made by the editors after a strict verification process.

In summary, the above-described investigation indicated that the peer reviews conducted for *FSR* since the journal’s establishment were not affected by any fake review/reviewers scams. The investigation yielded positive results, which foster our confidence in the integrity of *FSR*’s peer-review process. *FSR* and its editorial staff will continue to publish timely, innovative and high-quality papers and maintain the highest standards of peer review for every manuscript submitted to the journal.

Shiying Li *Editorial Office of Forensic Sciences Research, Key Laboratory of Forensic Medicine, Shanghai Forensic Service Platform, Academy of Forensic Science, Shanghai, China* lisy@ssfjd.cn Received 21 November 2019; accepted 2 December 2019
